# Differentially expressed proteins in plasma-derived extracellular vesicles from chronic myeloid leukemia patients

**DOI:** 10.3389/fgene.2026.1762244

**Published:** 2026-02-16

**Authors:** Denise Kusma Wosniaki, Alexandre Luiz Korte de Azevedo, Anelis Maria Marin, Alexis Germán Murillo Carrasco, Luciana Nogueira de Sousa Andrade, Rodrigo Brant, Michel Batista, Talita Helen Bombardelli Gomig, Roger Chammas, Mateus Nóbrega Aoki, Dalila Lucíola Zanette

**Affiliations:** 1 Laboratory for Applied Science and Technology in Health, Carlos Chagas Institute, Oswaldo Cruz Foundation (Fiocruz), Curitiba, State of Paraná, Brazil; 2 Department of Genetics, Federal University of Paraná, Curitiba, State of Paraná, Brazil; 3 Centro de Investigação Translacional em Oncologia (LIM24), Instituto do Câncer do Estado de São Paulo (ICESP), Faculdade de Medicina da Universidade de São Paulo (FMUSP), São Paulo, São Paulo, Brazil; 4 Comprehensive Center for Precision Oncology, Universidade de São Paulo, São Paulo, São Paulo, Brazil; 5 Mass Spectrometry Facility RPT02H, Carlos Chagas Institute, Oswaldo Cruz Foundation (Fiocruz), Curitiba, State of Paraná, Brazil

**Keywords:** biomarkers, chronic myeloid leukemia, extracellular vesicles, proteomics, T315I

## Abstract

**Introduction:**

Chronic myeloid leukemia (CML) is driven by the *BCR::ABL1* fusion gene. Although tyrosine kinase inhibitors (TKIs) have transformed outcomes, treatment resistance persists. Plasma extracellular vesicles (EVs) reflect their cell of origin and may serve as stable biomarkers.

**Methods:**

To characterize the plasma EV proteome in CML patients with distinct treatment responses and T315I mutation status. EVs were isolated from the plasma of healthy controls (HC) and CML patients classified as good (GTR) or poor (PTR) treatment responders, treatment-free remission (TFR), and T315I or pre-T315I mutation carriers. EVs were purified by size-exclusion chromatography, characterized by NTA and TEM, and analyzed by label-free mass spectrometry, followed by differential expression, enrichment, and protein–protein interaction analyses.

**Results:**

A total of 598 proteins were identified, 257 retained after quality and abundance filtering. Forty-two proteins were differentially expressed among HC, GTR, and PTR groups (p < 0.01), with PTR 27 samples showing marked downregulation of cytoskeletal and chaperone proteins (such as MYH9, 28 HSP90AB1, FERMT3). TFR patients exhibited distinct enrichment in complement and coagulation cascades (C3, C4B, F9, F11) and metabolic pathways.

**Discussion:**

Plasma EV proteomes reflect CML clinical status, revealing immune and cytoskeletal alterations associated with treatment response, remission, and resistance, suggesting potential biomarkers for disease monitoring.

## Introduction

According to estimates from the World Health Organization, leukemia accounted for approximately 486,777 new cases and 305,033 deaths globally in 2022, positioning the disease as the 13th most prevalent cancer and the 10th leading cause of cancer-related mortality (International Agency for Research on Cancer Who, 2022). Among leukemia subtypes, chronic myeloid leukemia (CML) is notably characterized by a reciprocal translocation between chromosomes 9 and 22, known as t (9; 22) (q34; q11) (Wosniaki et al., 2024). This chromosomal rearrangement generates the Philadelphia chromosome, containing the fusion of the *ABL1* gene on chromosome 9 (Murine Abelson) with the *BCR* gene (Breakpoint Cluster Region) on chromosome 22 (Luo et al., 2021). The consequent *BCR::ABL1* fusion transcript encodes a chimeric oncoprotein with constitutive tyrosine kinase activity, which aberrantly promotes signaling pathways that lead to leukemogenesis and uncontrolled proliferation of myeloid cells (Cumbo et al., 2020).

The advent of tyrosine kinase inhibitors (TKIs), such as imatinib, nilotinib, and dasatinib has significantly improved clinical outcomes by effectively targeting the *BCR::ABL1* kinase, reducing leukemic burden, resulting in increased number of patients in deep molecular response and remission (Jan et al., 2022). Despite these advances, the emergence of resistance to TKIs, which may result in suboptimal treatment response and disease progression to the accelerated or blast phase remains as a critical limitation (Osman and Deininger, 2021).

Molecular monitoring of *BCR::ABL1* transcript levels is fundamental to evaluate treatment efficacy and prognostic stratification in CML. Therapeutic resistance often arises from point mutations within the kinase domain of *BCR::ABL1*, which interfere with TKI binding and efficacy (Osman and Deininger, 2021). Molecular alterations have been evaluated as mechanisms of resistance to TKIs in CML, including mutations as T315I, Y253H, E255K/V, F359V and other mutations within the *ABL1* gene (Osman and Deininger, 2021). Alternative resistance mechanisms include activation of parallel survival and proliferation pathways, epigenetic alterations, and modifications in drug transport and metabolism, thereby adding complexity to disease management and reinforcing the importance of comprehensive molecular profiling to support the development of individualized treatment approaches (Osman and Deininger, 2021). However, despite advances in molecular diagnostics, the full spectrum of proteomic alterations associated with resistance phenotypes remains poorly characterized, underscoring the need for integrative approaches that capture post-transcriptional regulatory mechanisms.

Diverse biomarkers have been associated with both BCR::ABL1-dependent and -independent resistance mechanisms in CML, including coding and non-coding RNAs (ncRNAs) (Wosniaki et al., 2024). These biomarkers have potential for improving disease monitoring and may reflect dysregulated signaling pathways at various stages of CML progression. Previous findings from our group have identified mRNAs such as *PTGS2* and lncRNAs like *HOTAIR* as potential biomarkers associated with imatinib response in peripheral blood samples from CML patients (Kubaski Benevides et al., 2024; Wosniaki et al., 2025), as well as evidence of the regulatory role of miR-7-5p in CML plasma samples (Wosniaki et al., 2024).

Extracellular vesicles (EVs) have been described as active mediators in cancer, including hematological malignancies (Mutti et al., 2025). In chronic myeloid leukemia (CML), experimental studies have shown that leukemic cells release EVs capable of modulating components and of influencing signaling pathways associated with disease progression and response to tyrosine kinase inhibitors (TKIs) (Bernardi et al., 2024). Moreover, EV composition is influenced by the phenotypic or functional state of the producing cells (Bernardi et al., 2024). These observations provide a biological rationale to explore whether plasma-derived EV proteomes differ among CML patients with distinct treatment responses, and resistance-associated mutations.

Building upon these findings, the present study aimed to characterize the proteomic profile of plasma-derived extracellular vesicles (EVs) from CML patients at different stages of disease and therapeutic response, including the presence or absence of resistance mutations, these groups included patients with good progression and bad progression patterns under Imatinib treatment, and patients without resistance mutations, patients harbouring the T315I resistance mutation and patients who started a treatment free-remission protocol. Our approach provides a deeper understanding of the EV proteome and its potential contribution to find biomarkers of treatment response and resistance mechanisms in CML.

## Materials and methods

### Clinical samples analysis

Peripheral blood samples from CML patients were collected at the Erasto Gaertner Hospital (Curitiba, Brazil) following informed consent and approval from the local Ethics Committee (CAAE 08809419.0.0000.0098 and 53207021.5.0000.0098, issued on 23 May 2023). Plasma was first separated from the blood by centrifugation (400 g; 4 °C; 10 min) and carefully stored at −80 °C until use. The buffy coats were used to isolate DNA and RNA. Patients and samples were selected based on clinicopathological features (diagnosis, follow-up, prognosis, type and time of treatment, and presence/absence of resistance mutations).

The final cohort was composed of 5 groups related to the resistance-associated mutational burden of these patients (performed as indicated in the ‘Detection of *ABL1* mutations associated with TKI resistance’ section). These groups were called: PTR (patients with relapse but without resistance mutations, all those patients changed their treatment for dasatinib, n = 4); T315I (relapsed patients harboring T315I resistance mutation, which samples was also collected before the onset of the resistance mutation; samples named P-T315I, n = 4); GTR (patients that had a good prognosis evaluation, being treated with imatinib and a rapid decline of the *BCR::ABL1* transcript level, n = 4); TFR (patients who have responded well and started a discontinuation of the treatment, n = 4); and HC (samples from healthy blood donors, n = 5).

### Buffy coat RNA and DNA extraction

Two fractions of buffy coats were separated from peripheral blood samples; one fraction was used for total RNA extraction using the QIAamp® RNA Blood Mini Kit (Qiagen), while the other was used for DNA extraction with QIAamp® DNA Blood Mini Kit (Qiagen) following the manufacturer’s instructions. The extracted RNA and DNA were quantified in NanoDrop™ One (Thermo Scientific) and stored at −80 °C until further use.

### Quantification of *BCR::ABL1*

Total RNA extracted from the buffy coat was analysed by probe-based RT-qPCR following the protocol described by Marin et al. (2023). Briefly, reverse transcription and PCR amplification were performed as one-step reactions to evaluate the expression ratio between the *ABL1* endogenous gene and *BCR::ABL1* target transcriptional gene. Quantifications were based on a commercial standard curve, with the final results presented as the ratio of *ABL1* to *BCR::ABL1*, adjusted using a conversion factor and curve intersection values.

### Detection of *ABL1* mutations associated with TKI resistance

Specific Taqman™ SNP Genotyping Assays for each single nucleotide polymorphism (SNP) were used in qPCR reactions with TaqPath™ PromAmp™ Master Mix (Thermo Fisher Scientific). The PCR reactions were performed on a QuantStudio 5™ real-time PCR platform (Thermo Fisher Scientific) using the genotyping settings. The mutations that were characterized were: T315I (rs121913459), E255K (rs121913461), Y253H (rs121913461), E255V (rs121913449) and F359V (rs121913452), on all the analysed cohort just patients with the mutation T315I detected were allocated at the T315I group, there are no other patients with other mutations in any other groups.

### Extracellular vesicles (EVs) isolation

EV isolation and characterization were performed in accordance with current MISEV guidelines (Welsh et al., 2024), using complementary physical and morphological approaches.

EVs were isolated from 500 μL of plasma samples from CML patients using size-exclusion chromatography columns for extracellular vesicles 70 nm series (Izon Science Limited) following the recommendations of the manufacturer, no protein depletion step was applied during EV isolation, which was performed solely by size-exclusion chromatography, a technique known to preserve EV integrity. Fractions 1 and 2 were selected for downstream analyses as they are enriched in small extracellular vesicles according to the column specifications and are commonly used to minimize possible contaminations by soluble plasma proteins and lipoproteins. These fractions showed particle size distributions compatible with small EVs as assessed by nanoparticle tracking analysis (NTA).

### Nano particle tracking (NTA) analysis

Samples from the same analytic group were pooled and diluted 1:12 in filtered PBS (using 0.2 μm filters) to a final volume of 1 mL prior to analysis and quantification using a NanoSight LM14C instrument (Malvern Instruments, Malvern, United Kingdom), samples from the same analytical group were pooled in order to obtain sufficient particle concentration for reliable size distribution and concentration measurements. Measurements were performed at an optimal concentration of 20–100 particles per frame. Camera level was adjusted to clearly visualize all particles while maintaining particle signal saturation below 20% (for cell line-derived EVs, camera levels ranged between 14 and 15). The detection threshold was optimized to capture the maximum number of particles, typically aiming for 10–100 red crosses, while limiting blue crosses to a maximum of 8. Autofocus was fine-tuned to eliminate indistinct particles from the field of view.

For each sample, three videos of 60-s were recorded under the following conditions: temperature at 25 °C, infusion rate set to 1,000 units, and syringe pump speed at 100 μL/s. Captured videos were analyzed using the NanoSight Analytical Software (NTA 3.4, Build 3.4.4), applying a detection threshold of 2 or 3 to minimize the inclusion of non-EV particles.

### Transmission electron microscopy

The same sample’ pools that were used in the NTA analysis were visualized by Electron microscopy (pooled samples from each analytical group were used to allow representative visualization of vesicular structures). Each pool of EVs were loaded separately on 300-mesh Formvar-coated copper grids (Electron Microscopy Science, Washington, DC, United States of America) and allowed to adsorb for 1 h. The grids were then washed and prepared for the analysis as described by Souza et al. (2024). The analysis was carried out at the microscopy facility of the Carlos Chagas Institute (Oswaldo Cruz Foundation, Fiocruz- Paraná), performed at an electron microscope with acceleration voltage of 100 kV in a JEOL JEM-1400 Plus transmission (JEOL Ltd., Tokyo, Japan).

### EV proteins isolation and quantification

Protein contents were isolated from extracellular vesicles with a lysis buffer provided in the Total Exosome RNA and Protein Isolation Kit (ThermoFisher Scientific). Proteins were quantified using Qubit™ Protein and Protein Broad Range (BR) Assay Kits (ThermoFisher Scientific) in a Qubit fluorometer (ThermoFisher Scientific) and were sent to the mass spectrometry facility (Oswaldo Cruz Foundation, Fiocruz- Paraná).

### Depletion of high-abundance proteins from human plasma

The Multiple Affinity Removal Spin Cartridge Human-14 kit (Agilent) was used to deplete 14 plasma high-abundant proteins, including albumin, aiming to decrease sample complexity and improve detection sensitivity, high-abundance plasma protein depletion was carried out only after EV isolation, characterization by NTA and TEM, and vesicle lysis, and therefore could not affect EV recovery or structural integrity. The depletion process consisted of 3 stages: sample preparation, depletion by column affinity, and sample concentration. The Evs samples were centrifuged and applied to a 0.22 µm spin filter to remove debris and impurities that could clog the column. Then, 10 µL of the filtered sample was transferred to a new tube, and the depletion was realized following the kit standard pipeline, using a column affinity, resulting in 1 mL of depleted sample. After depletion, 500 µL were concentrated by Amicon ultra – 3 kDa, resulting in 200 µL of depleted and concentrated samples. This process was performed for all 28 plasma samples. The protein concentration was measured by UV 280 nm (ThermoFisher Scientific) before and after depletion and after the concentration step. The absorbance value was corrected using the molar absorption coefficient. Next, the samples were prepared for the NanoLC-MS/MS analysis.

### Sample preparation for NanoLC-MS/MS

Digestion of depleted Evs was carried out with 40 µg of proteins after sample drying in SpeedVac Vacuum Concentrator (ThermoFisher Scientific). Protein denaturation and reduction were achieved by sample incubation with 40 µL of urea 8 M in ammonium bicarbonate (ABC) 50 mM and DTT 25 mM for 1 h at 37 °C and 800 rpm followed by alkylation with 5.45 µL iodoacetamide 500 mM for 1 h at room temperature 25 °C and 800 rpm in the dark. Urea 8 M was diluted to 0.8 M with ammonium bicarbonate 50 mM prior to digestion with Sequencing Grade Trypsin (Promega) at 1:50 enzyme:protein m/m for 18 h at 37 °C. Before injection, samples were desalinated with an in-house stage-tip, and the peptides were quantified in NanoDrop (ThermoFisher Scientific) at 280 nm absorbance. Sample concentrations were normalized prior to injection.

### NanoLC-MS/MS analysis

The digested samples (0.5 µg) were separated by online nanoscale capillary liquid chromatography and analyzed by nanoelectrospray tandem mass-spectrometry (nLC-MS/MS). The chromatography was performed on an Ultimate 3,000 nanoLC (ThermoFisher Scientific) followed by nanoelectrospray ionization, and MS/MS on an Orbitrap Fusion Lumos (Thermo Fisher Scientific). The chromatographic conditions were as follows: mobile phase A 0.1% formic acid, mobile phase B 0.1% formic acid, and 95% acetonitrile. The flow of 250 nL/min, with a 60 min linear gradient from 5% to 40% B. The separation was carried out on an in-house C18 packed emitter with 15 cm length, 75 μm Internal diameter, packed with 3.0 μm C18 particles (Dr. Maisch - ReproSil-Pur). MS and MS/MS scan parameters were as follows: MS1 acquisition in the Orbitrap analyzer with a resolution of 120,000, m/z window of 300–1,500, positive profile mode with a maximum injection time of 50 ms. MS2 analysis was performed in data-dependent acquisition (DDA) mode of ions with 2-7 charges, 2 s per cycle where the most intense ions were subjected to high energy collisional dissociation (HCD) fragmentation at 30% normalized collision energy, followed by acquisition in the Orbitrap analyzer with a resolution of 15,000 in centroid mode. A dynamic exclusion list of 60 s and the internal mass calibration for the MS1 scans were applied. The nESI voltage was 2.3 kV, and the ion transfer capillary temperature was 275 °C.

### Raw proteomics data analysis

The spectra identification was carried out in MaxQuant (v. 2.2.0.0) as follows: Oxidation on methionine and acetylation on protein N-terminal were set as variable modifications, and carbamidomethylating of cysteine was selected as fixed modifications. The analysis was performed using the specific digestion mode and trypsin defined as protease. The search tolerance was set to 20 ppm for both MS and MSMS. The search was conducted using a peptide length of at least seven amino acids and a tolerance of two missed cleavages in the *in silico* digestion.

The *Homo sapiens* dataset was downloaded from UniProt (UP000005640; 81,837 entries) in January 2023 and used as the reference proteome, to which common contaminants (human keratins, BSA, and porcine trypsin) were added. The reverse dataset was employed as a decoy for FDR estimation (1% FDR for both Peptide Spectrum Match and protein assignment was accepted). The ‘match between runs’ and ‘label-free quantification’ (LFQ) options were enabled.

The mass spectrometry proteomics data has been deposited to the ProteomeXchange Consortium via the PRIDE25 (Project accession: PXD071200) partner repository.

### Protein expression data processing and analysis

All data processing was conducted at the Perseus software (v. 2.0.11) (Tyanova et al., 2016) using the Label-free quantification intensity (LFQ-intensity) to estimate protein expression levels. Initially, proteins indicated as potential contaminants, reverse hits, or only identified by site were excluded. To ensure data robustness, a filter was applied where only proteins detected in ≥70% of samples within at least one analytical group were retained for statistical analysis. LFQ-intensity values were then log_2_-transformed, and missing values were imputed from a normal distribution (width = 0.3; downshift = 1.8), to model low-abundance proteins below the detection limit and minimize bias introduced by stochastic MS sampling. Differential expression analyses were performed on continuous log_2_-transformed LFQ intensity values and did not rely on presence/absence-based protein detection.

Two statistical approaches were used to identify differentially expressed proteins (DEPs): Student's t-test (P-value <0.05) for two-group comparisons, and ANOVA followed by Tukey’s post-hoc test (FDR-value <0.05) for multiple-group comparisons. Principal component analysis (PCA) was performed in Perseus. Paired t-tests (P-value <0.05) were carried out using R software (v. 4.4.2) (https://www.R-project.org/).

### Functional enrichment analysis and protein-protein interaction network construction

Biological processes and pathways significantly enriched in the lists of differentially expressed proteins (DEPs) were identified using the REACTOME and Hallmark gene sets from the Molecular Signature Database (MSigDB; v. 2024.1) (Liberzon et al., 2015; Subramanian et al., 2005). Additionally, Gene Ontology (GO) terms related to cellular components and molecular functions were evaluated in the total proteome to verify whether the observed enrichment was consistent with known EV proteome characteristics. Gene Set Variation Analysis (GSVA) was performed using the GSVA R package (v. 2.0.7) (Hänzelmann et al., 2013) to calculate the activation scores for each sample based on the significantly enriched pathways. The kernel used for non-parametric estimation of the empirical cumulative distribution function (ECDF) across samples was set to ‘auto’ for optimal selection based on input data characteristics.

Protein–protein interaction (PPI) data was obtained from the STRING database (v. 12.0), incorporating experimentally validated interactions, co-expression data, and data from partner databases (Szklarczyk et al., 2021). Interactions were categorized as medium-confidence (score 0.4–0.7) or high-confidence (score >0.7). The resulting networks were visualized using Cytoscape software (v. 3.10.3) (Shannon et al., 2003).

The total proteome obtained after the filtering stages was compared to the Vesiclepedia (Kalra et al., 2012) and ExoCarta (Keerthikumar et al., 2016) annotated proteomes to verify if our data was enriched in previously EV-associated proteins. The 100 most often identified proteins in both databases were considered as EV-markers in this analysis.

## Results

### Cohort characterization

All the selected patients were diagnosed with CML, and samples were selected using the criteria of a time collection by 20 months (±6) after the initiation of treatment (patients with resistance mutations were selected based on the mutation point). In this way, the selected groups are demonstrated at Table 1 and the clinical information with the *BCR::ABL1* quantification and mutational profile are demonstrated in the Supplementary Table S1.

**TABLE 1:** Cohort description demonstrating the group selection, therapeutic intervention and treatment.

Analytic group	Number of samples	Therapeutic intervention	Treatment
Poor treatment response (PTR)	4	Patients who switched to dasatinib following imatinib failure and relapse. Absence of resistance mutations	Dasatinib for 20 months ±6
Good treatment response (GTR)	4	Imatinib-treated patients with rapid *BCR::ABL1* decline and favorable prognosis	Imatinib for 20 months ±6
Treatment-free remission (TFR)	4	Patients who responded well and initiated treatment discontinuation	20 months ±6 after stopping the treatment with Imatinib
T315I carriers (T315I)	4	Patients evaluated in paired samples at the time of T315I resistance mutation detection, presenting relapse and confirmed mutation status	Variable, different treatments and time lapses were observed and the selected sample were at the moment where the mutation was detected
Pre-T315I mutation (P-T315I)	4	Patients evaluated in paired samples approximately 12 ± 4 months prior to the detection of the T315I resistance mutation, before clinical relapse	Variable, different treatments and time lapses were observed and the selected sample were approximately 12 ± 4 months prior to the detection of the mutation
Healthy control (HC)	5	Samples obtained from healthy control donors	Not applicable

### Extracellular vesicles characterization

**FIGURE 1:**
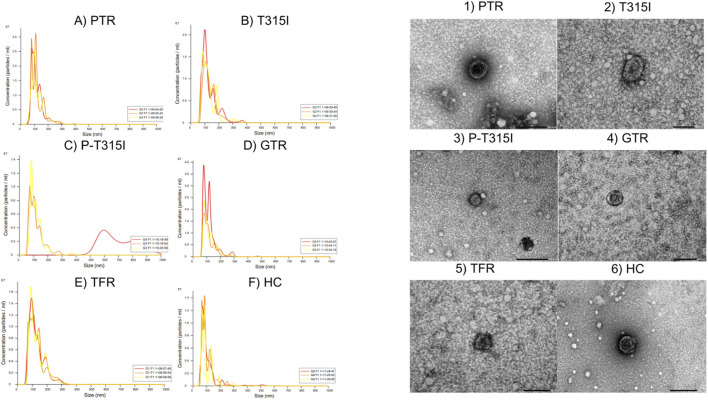
**(A–F)** Nanosight results showing EVs concentration and size for 3 recorded times for each group and 1–6) Electron microscopy demonstrating the incidence of EVs on the pools of samples. PTR: Group of patients that had a relapse after the treatment with Imatinib; T315I: Patients positive for the T315I resistance mutation; P-T315I: Group of patients that had the T315I resistance mutation, but in a point point previous to the acquisition of the mutation; GTR: Group of patients with a good progression in the treatment with Imatinib; TFR: Patients with a very good progression in the treatment that started a free-remission treatment; HC: Health control group.

The EV isolation protocol resulted in 5 EV size fractions: Fraction 1 is enriched in smaller extracellular vesicles, while fraction 5 is enriched in larger vesicles. The fractions 1 and 2 (smaller EV fractions) from each sample, were mixed and the size distribution confirmed by Nanosight tracker (NTA) and Electron microscopy, where the samples within the same group were pooled and analysed as an unique sample (Figure 1; Supplementary Table S1), demonstrating the presence of EVs in each of these pools. For MS analyses, patients were analysed individually were each sample was composed by the mix between fractions 1 and 2, samples were prepared for MS analyses, to assess the differentially expressed proteins in each sample from the analytical groups. NTA demonstrated particle size distributions consistent with small extracellular vesicles across all pooled samples. TEM analysis further confirmed the presence of membrane-bound vesicular structures with sizes compatible with EVs.

### Proteomic evaluation of CML extracellular vesicles

**FIGURE 2:**
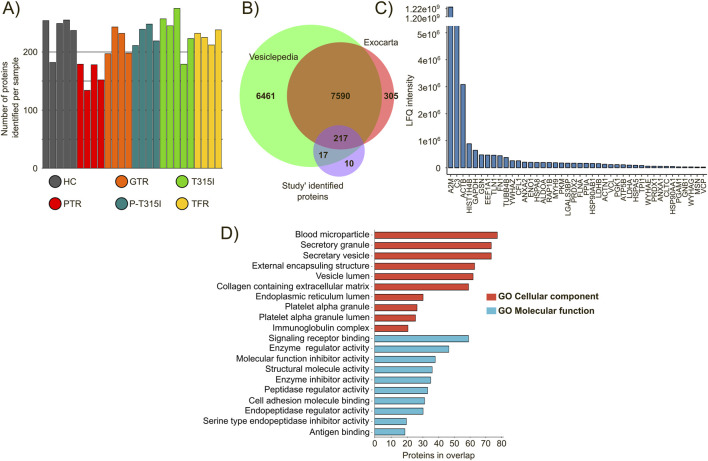
Characterization of the differentially expressed proteins identified in chronic myeloid leukemia extracellular vesicles across analytical groups. **(A)** Number of proteins identified across samples (FDR 1%). **(B)** Correspondence of the identified proteome (257 proteins) with the annotated extracellular vesicles proteomes annotated in the Vesiclepedia and ExoCarta databases. **(C)** EV-associated protein markers identified in the study according to the top100 most identified EV-proteins in the Vesiclepedia and ExoCarta databases. **(D)** Gene Ontology enrichment of cellular components and molecular functions of the identified EV proteome (FDR <0.05).

After EV isolation, their protein contents were released, prepared and analyzed by a high-throughput label-free mass spectrometry approach, resulting in the identification of 598 proteins across samples at FDR 1% (mean 214.96 proteins per sample) (Figure 2A; Supplementary Table S2). To obtain a more representative proteome, we filtered the identified proteins to retain only those detected in a minimum of 70% of the samples within at least one analytic group, resulting in 257 proteins selected for downstream analysis (Supplementary Table S2). To assess whether the identified proteomic profile was consistent with the canonical content of human extracellular vesicles, we compared our protein list with the annotated proteome found in Vesiclepedia and ExoCarta databases. As shown in Figure 2B, only 10 proteins found here were not previously annotated in extracellular vesicles (Supplementary Table S3). Apolipoproteins represented 12 out of the 258 (4.6%) total identified proteins after filtering (Supplementary Table S2), which indicates a small percentage of contamination with plasma proteins.

Notably, the identified proteome included classic EV-associated proteins such as the chaperones HSP90AA1, HSP90AB1, HSPA5, and HSPA8 as well as A2M, C3, ANXA1, ANXA2 (Figure 2C). Additionally, we performed functional enrichment analysis using GO terms, identifying cellular compartments and molecular functions that were consistent with those expected for the EV proteome (Figure 2D), such as blood microparticles, vesicle lumen and secretory granules and vesicles.

To determine whether the total proteome was able to segregate the groups of samples, a PCA and an unsupervised hierarchical clustering (Supplementary Figure S1A,B) were performed. No clear separation of the groups could be observed, which highlights the subtle differences between the groups of patients and the challenges to identify differentially expressed proteins across groups to better capture the proteomic signatures associated with distinct clinical and biological behaviors in CML.

### Proteome profiles can be associated with the biological and clinical behavior in patients with different treatment response levels

**FIGURE 3:**
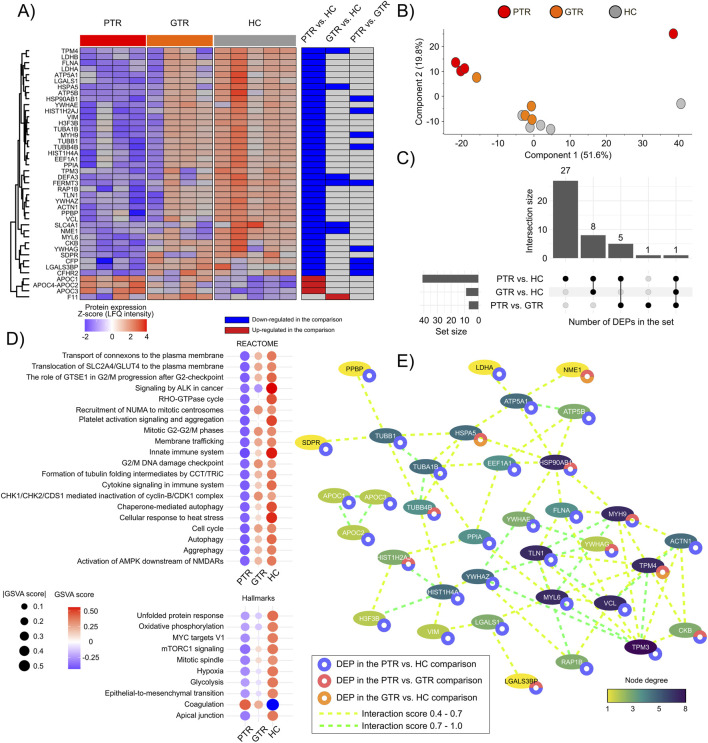
Identification and functional analysis of differentially expressed EV proteins identified in non-tumor and tumor samples with different prognosis. **(A)** The heatmap illustrates the expression patterns of the 42 DEPs identified in the comparison of healthy control samples and poor and good prognostic samples (ANOVA-Tukey post-hoc test, P-value <0.05). **(B)** PCA plot of the sample’s segregation according to the expression of the 42 DEPs. **(C)** Distribution of the identified DEPs across comparison pairs. **(D)** Functional enrichment analysis of the REACTOME and hallmark collections of MSigDB (v. 2024.1) (FDR <0.05). The circle’s color and size indicate the relative activation GSVA scores associated with each process across sample groups. **(E)** Protein-protein interaction network of the identified DEPs. Proteins without interaction partners are omitted. The node’ color indicates the degree of each protein in the PPI.

To gain a broader understanding of how the EV-proteome may impact the response to treatment in CML patients, the EV-proteomes of the good response (GTR), poor response (PTR) and healthy controls (HC) groups were compared (FDR <0.05), which led to the identification of 42 DEPs (Figure 3A; Supplementary Table S2). In the PCA analysis, PTR samples segregated from the HC and GTR samples, which clustered closely together (Figure 3B). The distribution of DEPs across comparisons is illustrated in Figure 3C. Most DEPs showed a decreased expression in the PTR group when compared to the HC samples. Additionally, DEPs such as DEFA3, FERMT3, TPM4, HSPA5, SLC4A1, and NME1 were downregulated in CML x HC samples independently of the treatment response level.

The functional enrichment and GSVA analyses (FDR value <0.05; Supplementary Table S4), allowed the identification of significantly enriched cancer-related pathways, including cell cycle, autophagy, glycolysis, aggrephagy, hypoxia, epithelial-to-mesenchymal transition (EMT), and cytokine signaling in the immune system. Key proteins involved in these processes included the tubulin isoforms TUBA1B, TUBB1, and TUBB4B, as well as the tyrosine 3-monooxygenases YWHAE, YWHAG, and YWHAZ, along with FLNA, VIM, HSPA5, and LDHA (Figure 3D). Other processes included Rho-GTPases, mTORC1, and ALK signaling pathways (MYH9, MYL6, VCL, TPM3, and TPM4), chaperone-mediated autophagy (EEF1A1, HSP90AB1, and VIM), and oxidative phosphorylation (LDHA, LDHB, ATP5A1, ATP5B). Notably, all these processes showed higher GSVA scores in HC samples compared to CML samples, with the lowest GSVA scores observed in the PTR samples, suggesting a decline in the expression of these EV proteins in samples with a poor response to leukemia treatment.

A protein-protein interaction (PPI) analysis demonstrated a significant PPI enrichment (PPI enrichment score = 1.11e-16) associated with the 42 DEPs. The most interconnected DEPs in the PPI were VCL, TMP3, TPM4, TLN1, HSP90AB1, and MYH9 (Figure 3E; Supplementary Table S5).

### EVs proteomic profiles can reflect CML clinical particularities

Following the initial assessment of proteome profiles in EVs from HC samples and CML samples with distinct treatment responses, we extended the analysis to include the T315I and TFR groups. This approach deepens the evaluation of proteomic alterations across distinct prognostic profiles, capturing the potential impact of the T315I mutation and proteomic particularities associated with treatment-free remission events.

**FIGURE 4:**
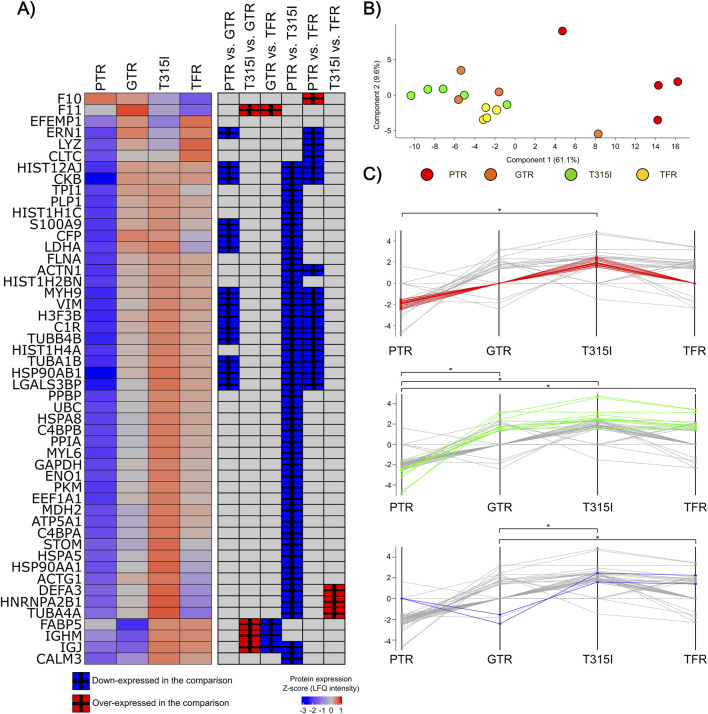
EV-protein expression alterations associated with distinct clinical characteristics across CML patient groups. **(A)** Heatmap of the 50 DEPs identified across groups (FDR <0.05) and the comparisons in which significant differences were observed (Tukey post-hoc test). **(B)** PCA analysis of the samples’ distribution according to the 50 DEPs expression. **(C)** Profile-plots indicating protein clusters associated with different sample groups. The profile plots are constructed using the mean expression of each DEP in the sample groups. A p-value of <0.05 was considered significant. *: p < 0.05, **: p < 0.01, **: *p < 0.001, ****: p < 0.0001. ns: non-significant.

We identified 50 DEPs across groups (FDR <0.05; Supplementary Table S2), with the majority observed in the PTR samples compared to the other groups (Figure 4A). Consistently, the PCA analysis showed a clear separation of PTR samples, while the remaining groups clustered together (Figure 4B). A cluster of 23 DEPs, including FLNA, HSPA5, HSPA8, MDH2, PKM, and STOM, presented a significant lower-expression in the PTR group compared to the T315I patients, while a 10-DEP cluster, including DEPs such as C1R, VIM, CKB, TUBA1B and TUBB4B, presented lower-expression in PTR compared to all other sample groups simultaneously. Also, a cluster composed by FABP5 and IGHM presented lower expression in the GTR samples compared to the TFR and T315I patients (Figure 4C).

### Treatment-free remission patients present a distinct EV protein expression pattern

To better elucidate if proteomic alterations are associated with the treatment remission profile in chronic myeloid leukemia, we compared the proteomes of TFR, good (GTR) and poor treatment (PTR) response groups.

**FIGURE 5:**
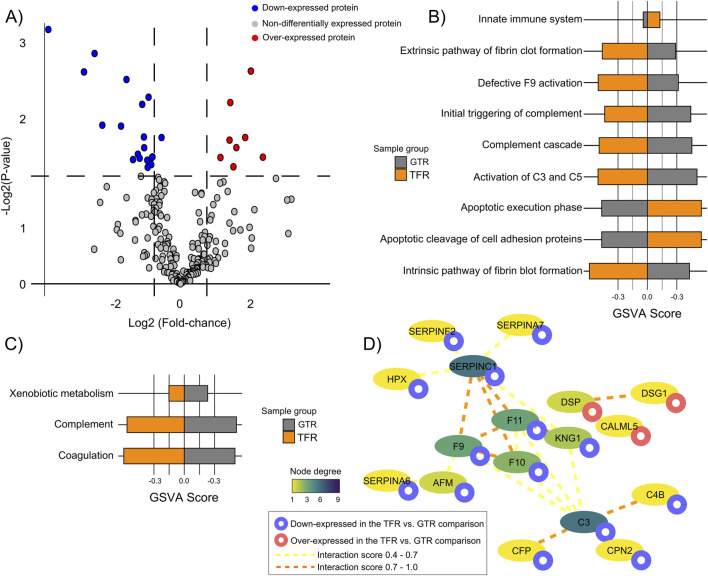
Functional and interactome profiles of differentially expressed proteins (DEPs) in treatment-free remission (TFR) versus good treatment response (GTR) patients. **(A)** Number of DEPs identified in the TFR vs. GTR comparison (non adjusted P < 0.05). **(B)** REACTOME pathways and GSVA scores associated with DEPs from the TFR vs. GTR comparison (FDR <0.05). **(C)** Hallmarks and GSVA scores associated with DEPs from the TFR vs. GTR comparison (FDR <0.05). **(D)** PPI network of DEPs from the TFR vs. GTR comparison. Proteins without interaction partners are omitted. The node’s color indicates the degree of each protein in the PPI.

The comparison of TFR and GTR revealed 29 DEPs (Figure 5A; Supplementary Table S2). IGKV1-5, CFP, C3, C4B, and CPN2 were enriched in the innate immune-system, complement cascade, and neutrophil degranulation processes; DSG1 and DSP, which were associated with apoptotic execution phase and cleavage of adhesion proteins, and F10, F11, ARG1, and SERPINA6, which were enriched in xenobiotic metabolism (Figures 5B,C; Supplementary Table S4). The TFR x GTR PPI network (PPI enrichment score <1.0e-16) presented C3, SERPINC1, F9, and F11 as the more interconnected nodes (Figure 5D; Supplementary Table S5).

**FIGURE 6:**
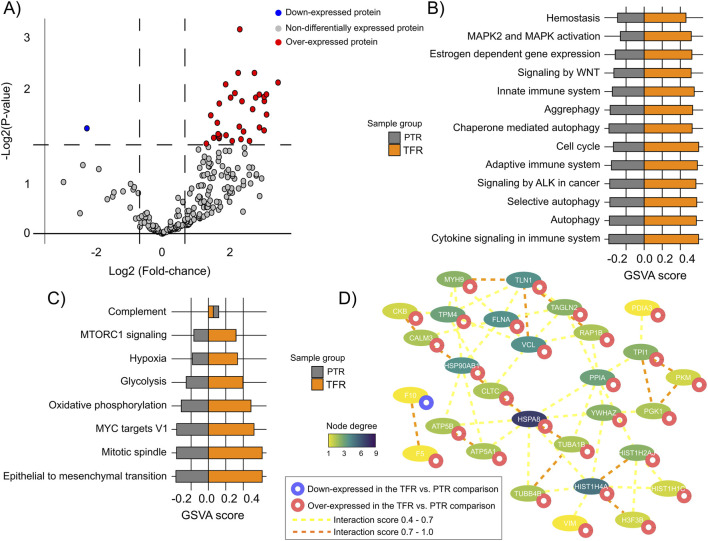
Functional and interactome profiles of differentially expressed proteins (DEPs) in treatment-free remission (TFR) versus poor treatment response (PTR) patients. **(A)** Number of DEPs identified in the TFR vs. PTR comparison (P < 0.05). **(B)** REACTOME pathways and GSVA scores associated with DEPs from the TFR vs. PTR comparison (FDR <0.05). **(C)** Hallmarks and GSVA scores associated with DEPs from the TFR vs. poor response comparison (FDR <0.05). **(D)** PPI network of DEPs from the TFR vs. PTR comparison.

By comparing TFR and PTR samples we identified 37 DEPs, with prevalence of over-expressed DEPs in the TFR group (Figure 6A; Supplementary Table S2). These proteins were enriched in processes such as MAPK2 and MAPK activation (VCL, TLN1, and RAP1B), hypoxia and glycolysis (PGK1, TPI1, PPIA, and PKM), aggrephagy and autophagy (TUBAB1, TUBB4A, VIM, HSPA8, HSP90AB1), cell cycle (YWHAZ, H4C1, H2AC14, H3-3B), and signaling by WNT, MTORC1, and ALK (PGK1, TPI1, PPIA, YWHAZ, CLTC, TPM4). The TFR x PTR PPI network (PPI enrichment score = 6.25e-11) included chaperones such as HSP90AB1, HSPA8, and HSPA5, besides H4C6, VCL, TPM4, and TUBA1B as its more interconnected nodes (Figure 6D; Supplementary Table S5).

DNA and histone methylation processes and chromatin modification (H3-3B, H2BC15, H4C1, H2AC14), KRAS and PI3K-AKT-mTOR signaling (F13A1, CFHR2, PPBP, CLTC, CFL1), as well as apical junctions and GAP-junction trafficking (MYH9, VCL, TUBB4A, TUBA1B, TUBB1) (Figures 6B,C; Supplementary Table S4) were enriched in TFR x PTR. The TFR x PTR PPI network (PPI enrichment score = 5.77e-15) included chaperones such as HSP90AB1 and HSPA8, besides FLNA, VCL, TLN1, and HIST1H4A as its more interconnected nodes (Figure 6D; Supplementary Table S5).

### Effect of T315I mutation on the EV-proteome of chronic myeloid leukemia patients

**FIGURE 7:**
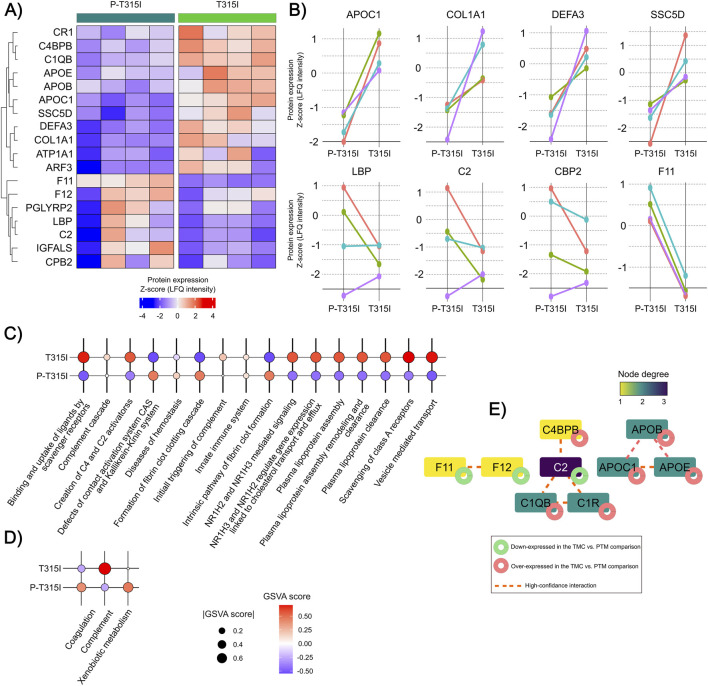
Expression analysis of EV proteome of patients before and after acquisition of the T315I mutation. **(A)** Heatmap illustrating the expression pattern of the DEPs identified in the comparison (Paired t-test; P-value <0,05). **(B)** Line plots depicting the expression dynamics of the four most over-expressed DEPs and the four more down-expressed DEPs after T315I mutation acquisition. **(C)** REACTOME pathways and GSVA scores associated with the DEPs from the TMC vs. PTM comparison (FDR <0.05). **(D)** Hallmarks and GVA scores associated with the DEPs from the TMC vs. PTM comparison (FDR <0.05). **(E)** Protein-protein interaction network of the DEPs identified in the TMC vs. PTM comparison. Green circles = down-expressed DEPs. Red circles = over-expressed DEPs. Proteins without interaction partners are omitted. The node’ color indicates the degree of each protein in the PPI.

In order to determine the impact of the T315I mutation on the EV-proteome, we compared the proteome of paired patients before and after acquisition of the mutation. As shown in Figure 7A, we identified 17 DEPs (Supplementary Table S2). APOC1, COL1A1, DEFA3, and SSC5D presented the most conspicuous expression increase after mutation acquisition, while LBP, C2, CBP2, and F11 the most evident expression decrease after mutation (Figure 7B).

These DEPs were enriched in processes such as scavenging receptor functioning (COL1A1, APOE, SSC5D), NR1H2 and NR1H3 signaling (APOE, APOC1), plasma lipoprotein clearance (APOE, APOC1), and complement cascade (C1QB, C1R, C2, CPB2,C4BPB), among others (Figures 7C,D; Supplementary Table S4). The PPI network associated with these DEPs presented a significant PPI enrichment score (PPI enrichment score = 6.97e-07) (Figure 7E; Supplementary Table S5).

## Discussion

This report shows the differentially expressed proteins found in plasma EVs obtained from healthy and CML individuals, the latter group subdivided according to disease stage and therapy response/resistance status. To our knowledge, the only similar report described the plasma EV proteome of 18 CML patients, 9 imatinib sensitive and 9 imatinib resistant, regardless of the presence of ABL1 mutations and did not include patients in TFR. In this previous report, the functional analysis showed upregulated and downregulated proteins that were related to ribosome and lipid metabolism, respectively (Li et al., 2021). By comparing those results with the present, little similarity was found, as the current analysis relied on additional factors and different comparisons, instead of being limited to imatinib resistance. In the current report, a group of healthy individuals was included, as well as subgroups of patients subdivided to disease and resistance status: GTR, PTR, patients with the T315I mutation, as well as patients in TFR.

The comparison between HC, PTR and and GTR and further selection of the DEPs found in PTR vs. GTR, showed HSP90AB1, HIST1H2AJ, MYH9, TUBB1, FERMT3, YWHAG, CFP, LGALS3BP and CFHR2 as downregulated proteins in EVs from PTR compared to GTR.

HSP90 is one of the major intracellular molecular chaperones from the Heat shock proteins (HSP) family, which interacts with various intracellular proteins to ensure their correct folding and function. HSP90AB1, also recognized as HSP90 beta, is commonly found in EVs (Ono et al., 2018) and its role stabilizing oncoproteins to inhibit apoptosis was described in some tumors including leukemias. It was previously reported that Hsp90 protein levels correlate with good and poor responses to CML therapy, as leukocytes from patients in major molecular response (MMR) had low expression levels of Hsp90, similar to those in healthy individuals, while increased Hsp90 levels were found in patients with resistance to therapy and in hematological relapse (Záčková et al., 2013). It has also been shown that HSP90AB1 interacts with BCR-ABL, preventing its transport to the nucleus while inhibition of HSP90AB1 induces nuclear localization of Bcr/Abl and results in apoptosis of CML cells (Peng et al., 2021). These previous findings seem opposite to our results in EVs, in which lower levels of HSP90AB1 were found in EVs from poor responders. However, an important finding from Ono and colleagues showed that the protein levels in the EV are not necessarily predicated by their relative intracellular concentrations (Ono et al., 2018). Also, our study focused on proteins found within EVs, which correspond to a small fraction of the total of proteins that can be found circulating in the blood. Hence, HSP90 may be present in its soluble isoform or may be carried by other types of vesicles that were not analyzed in the present study. The role of HSP90 proteins in extracellular vesicles may be broader than currently known, as extensively reviewed by Egushi and colleagues (Eguchi et al., 2019) or, alternatively, these proteins may simply act to help the transport of their client proteins. MYH9 is also an exosome-related protein/gene whose up-regulation has been described in K562 cell lines treated with Imatinib and Nilotinib (Hekmatshoar et al., 2024), and as a target of TKIs. In our data, it is interesting to notice the lower expression of MYH9 in PTR, while GTR, TFR patients and healthy controls had higher levels of MYH9, implicating a possible role for this protein in the poor response to Imatinib. FERMT3 regulates c-Myc protein expression in the human CML cell line K562 (Qu et al., 2015) but to our knowledge there are no reports on its expression in CML clinical samples. Despite this, an important observation came from the study of Tang and colleagues, which describes a decreased FERMT3 expression in colorectal cancer tissues and cell lines. The induced overexpression of FERMT3 inhibited CRC cell invasion, enhanced CRC sensitivity to 5-FU and suppressed PD-L1 via the blockage of the Wnt/β-catenin signallin, inducing NK cell-mediated tumour toxicity and hence, immune evasion in CRC (Tang et al., 2022). These effects of FERMT3 on PD-L1 may be especially important, since the inhibition of the PD-1/PD-L1 axis is an established anti-leukemic strategy, with an ongoing trial investigating the combination of pembrolizumab (PD-L1 antibody) with TKIs in CP-CML. Other means of inhibition may counteract PD-L1 activity, among other types of PD-L1 modulation (Patterson and Copland, 2023). Furthermore, the immune system is crucial to control residual leukemia cells in CML patients that maintain TFR, in which NK cells exert a pronounced role (Huuhtanen et al., 2024; Sanch et al., 2023). Based on these reports, it is possible to hypothesize that the down-regulation of FERMT3 EVs from CML poor responders could be related to a role of FERMT3 in the inhibition of PD-L1, which in turn, is involved in the optimal response to treatment. LGASL3BP, another down-regulated protein in poor responders, has been also described to be involved in PD-L1, as a combination therapy of anti-LGALS3BP with anti-PD-1 has resulted in a higher inhibition of tumor growth and prolonged survival in neuroblastoma mouse model (Cela et al., 2025).

The complement factor P (CFP or properdin) is the only known positive regulator of the alternative complement pathway and thus it may be linked to complement-dependent elimination of unwanted cells, such as cancer cells (Chen et al., 2018). To our knowledge, there are no previous reports of differential expression of CFP in CML patients, but increased levels of C5, C4 and inhibitory factor B occurred in the preceding time points in patients with optimal responses to treatment with TKIs (Janowski et al., 2024). CFHR2 is a regulator that inhibits the complement C3 alternative pathway (Jiang et al., 2025).

Complement components and coagulation factors were found differentially represented not only in PTR vs. GTR, but also in patients before and after T315I (P-T315 vs. T315), TFR vs. PTR and TFR vs. GTR. It is a very interesting result, with DEPs related to complement and coagulation pathways. Janowski and collaborators described a higher serum concentration of C1q, C4 and C5a 3 months after TKI treatment in CML patients who achieved optimal responses in the 6 months after diagnosis (Janowski et al., 2024). In TFR vs. GTR, C3 and C4B were down-regulated in TFR, as well as coagulation factors F9, F10 and F11. In this comparison, we also found four down-regulated SERPIN proteins in TFR: SERPINA6, SERPINA7, SERPINC1 and SERPINF2 and one up-regulated (SERPINB12). F10 was also found to be down-regulated in TFR vs. PTR, while F5 was up-regulated in TFR. No SERPINs and complement components were differentially expressed in TFR vs. PTR. In the cases before the acquisition of T315I (P-315I), complement components C2, CPB2 were down-regulated, while C1R, C4BPB and C1QB were up-regulated in P-T315I vs. T315I. Coagulation factors F11 and F12 were down-regulated in P-T315I. These different regulations of complement components may indicate a decreased complement activity in TFR and before the acquisition of T315I, mainly because of the down-regulation of the complement regulatory components, such as CBP2 and CFP (Ieko et al., 2010). A number of studies report the regulation of EV shedding and activation by complement system, as reviewed by Karasu and colleagues (Karasu et al., 2018). Although it is difficult to address a functional role for these DEPs in the EV from CML patients, it is plausible to hypothesize that the downregulation of complement components might influence immune response, especially in TFR patients and TKI good responders has been correlated not only to cell-mediated immune response (Hughes et al., 2017), but also to humoral immunity (Janowski et al., 2024), albeit in a lesser extent.

This study has limitations inherent to plasma-derived EV analyses. Although EV characterization was supported by SEC isolation, NTA, TEM, and enrichment of canonical EV-associated proteins in the proteomic dataset, molecular validation of EV markers (e.g., by Western blotting) was not performed. This limitation should be considered when interpreting the results and will be addressed in future studies. Given the limited number of samples per group (n = 4 per group), this study should be regarded as exploratory and hypothesis-generating. Validation in larger, independent cohorts will be necessary to confirm the robustness and clinical relevance of the identified EV-associated proteomic signatures.

To our knowledge, this is the only report showing differentially expressed proteins in plasma EVs from CML clinical samples, isolated from patients in distinct levels of response to therapy. The majority of reports are based on EVs from CML cell lines, making this a strength of the current report. Moreover, here groups of patients harboring T315I were also evaluated separately. As a weakness, a limited number of samples was evaluated. Nevertheless, a relevant number of DEPs were described in each group of patients, with special emphasis to those DEPS found in T315I patients and for the presence of complement components and coagulation factors.

## Data Availability

The datasets presented in this study can be found in online repositories. The names of the repository/repositories and accession number(s) can be found below: https://www.ebi.ac.uk/pride/archive/, PXD071200.
