# Supplementary material for: Differentially expressed proteins in plasma-derived extracellular vesicles from chronic myeloid leukemia patients
Source: Front Genet. 2026 Feb 16;17:1762244. doi: 10.3389/fgene.2026.1762244 (PMC12950269; doi:10.3389/fgene.2026.1762244)
Supplement: Supplementary file 3 [file DataSheet1.pdf]

Supplementary Material

1. Supplementary Figure 1

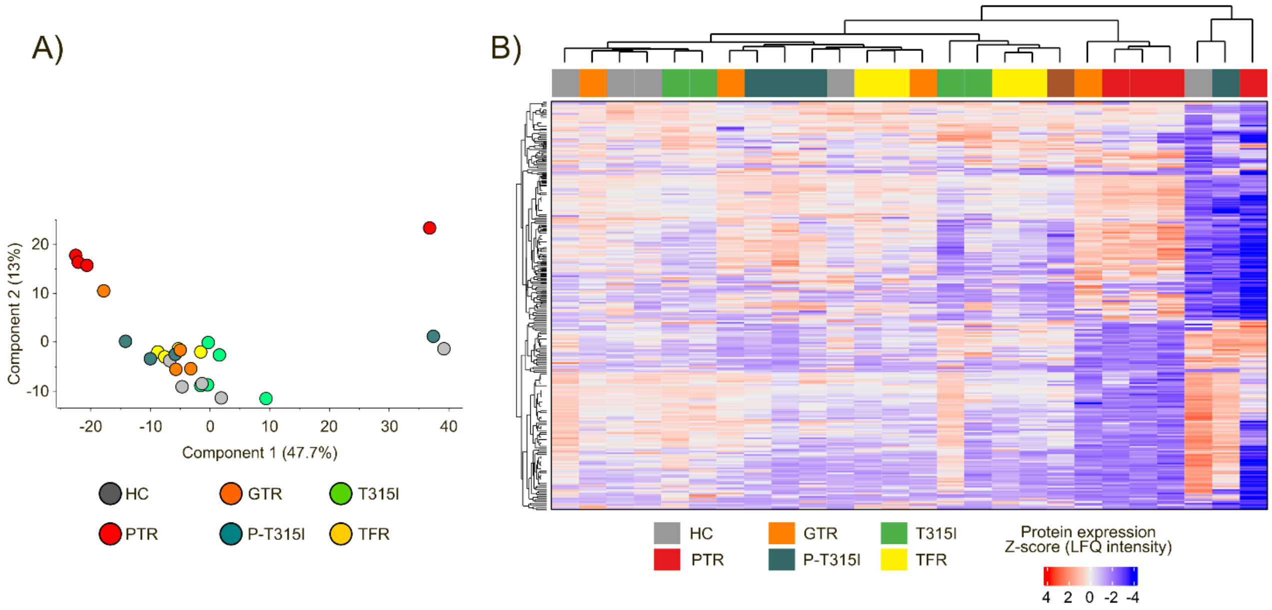

Supplementary Figure 1. Total proteome analysis demonstrating the segregation of the groups of samples, PCA and an unsupervised hierarchical clustering were applied.
